# A *GP1BA* Variant in a Czech Family with Monoallelic Bernard-Soulier Syndrome

**DOI:** 10.3390/ijms23020885

**Published:** 2022-01-14

**Authors:** Magdalena Skalníková, Kateřina Staňo Kozubík, Jakub Trizuljak, Zuzana Vrzalová, Lenka Radová, Kamila Réblová, Radka Holbová, Terézia Kurucová, Hana Svozilová, Jiří Štika, Ivona Blaháková, Barbara Dvořáčková, Marie Prudková, Olga Stehlíková, Michal Šmída, Leoš Křen, Petr Smejkal, Šárka Pospíšilová, Michael Doubek

**Affiliations:** 1 Center of Molecular Medicine, Central European Institute of Technology, Masaryk University, 625 00 Brno, Czech Republic; katerina.stanokozubik@ceitec.muni.cz (K.S.K.); trizuljak@gmail.com (J.T.); zuzana.vrzalova@ceitec.muni.cz (Z.V.); avodar@gmail.com (L.R.); kamila.reblova@ceitec.muni.cz (K.R.); radka.holbova@ceitec.muni.cz (R.H.); terezia.kurucova@ceitec.muni.cz (T.K.); svozilova.hana@gmail.com (H.S.); jiri.stika@ceitec.muni.cz (J.Š.); ivona.blahakova@ceitec.muni.cz (I.B.); michal.smida@ceitec.muni.cz (M.Š.); sarka.pospisilova@ceitec.muni.cz (Š.P.); 2 Department of Internal Medicine, Hematology and Oncology, University Hospital Brno and Faculty of Medicine, Masaryk University, 625 00 Brno, Czech Republic; dvorackova.barbara@fnbrno.cz (B.D.); stehlikova.olga@fnbrno.cz (O.S.); 3 Institute of Medical Genetics and Genomics, University Hospital Brno and Faculty of Medicine, Masaryk University, 625 00 Brno, Czech Republic; 4 Department of Clinical Hematology, University Hospital Brno and Faculty of Medicine, Masaryk University, 625 00 Brno, Czech Republic; prudkova.marie@fnbrno.cz (M.P.); smejkal.petr@fnbrno.cz (P.S.); 5 Department of Laboratory Methods, Faculty of Medicine, Masaryk University, 625 00 Brno, Czech Republic; 6 Department of Pathology, University Hospital Brno and Faculty of Medicine, Masaryk University, 625 00 Brno, Czech Republic; kren.leos@fnbrno.cz

**Keywords:** Bernard-Soulier syndrome, monoallelic, autosomal dominant, *GP1BA*, macrothrombocytopenia

## Abstract

Bernard-Soulier syndrome (BSS) is a rare inherited disorder characterized by unusually large platelets, low platelet count, and prolonged bleeding time. BSS is usually inherited in an autosomal recessive (AR) mode of inheritance due to a deficiency of the GPIb-IX-V complex also known as the von Willebrand factor (VWF) receptor. We investigated a family with macrothrombocytopenia, a mild bleeding tendency, slightly lowered platelet aggregation tests, and suspected autosomal dominant (AD) inheritance. We have detected a heterozygous *GP1BA* likely pathogenic variant, causing monoallelic BSS. A germline *GP1BA* gene variant (NM_000173:c.98G > A:p.C33Y), segregating with the macrothrombocytopenia, was detected by whole-exome sequencing. In silico analysis of the protein structure of the novel GPIbα variant revealed a potential structural defect, which could impact proper protein folding and subsequent binding to VWF. Flow cytometry, immunoblot, and electron microscopy demonstrated further differences between p.C33Y *GP1BA* carriers and healthy controls. Here, we provide a detailed insight into its clinical presentation and phenotype. Moreover, the here described case first presents an mBSS patient with two previous ischemic strokes.

## 1. Introduction

Bernard-Soulier syndrome (BSS) is a rare inherited platelet disorder characterized by abnormally large platelets—macrothrombocytes—thrombocytopenia, and prolonged bleeding time, as well as with failure platelet aggregation after ristocetin [1,2,3]. BSS is caused by mutations in one of the following genes: *GP1BA*, *GP1BB*, or *GP9*. The mode of inheritance is usually AR (biallelic)—classical BSS. Clinically, patients with classical BSS present with a severe bleeding tendency, especially after events such as childbirth or surgery. Heterozygous carriers are usually asymptomatic and have normal platelet counts, although some might have slightly enlarged platelets and decreased platelet glycoprotein GPIb-IX-V complex expression, as well as a moderately reduced Ristocetin-Induced Platelet Aggregation (RIPA) response, a test triggering the binding of VWF to GPIbα [4,5]. Moreover, rare variants in the *GP1BA* and *GP1BB* genes have been reported in patients with macrothrombocytopenia and the AD (monoallelic) mode of inheritance, presenting with a mild, usually asymptomatic form [3,4,5,6,7,8,9,10,11,12]. Around 12 missense mutations in the *GP1BA* gene have been described so far [4,8,9,10,11,12,13,14,15], one in a family from the Czech Republic. In addition, fourteen missense mutations in the *GP1BB* gene have been detected [2,4,5,6,10]. As GPIbα binds with other protein partners and influences primary hemostasis through several mechanisms, functional analysis of the aberrant protein is essential for confirmation of its pathogenicity [3,4,5,6].

## 2. Results

### 2.1. Clinical Phenotype

**Figure 1:**
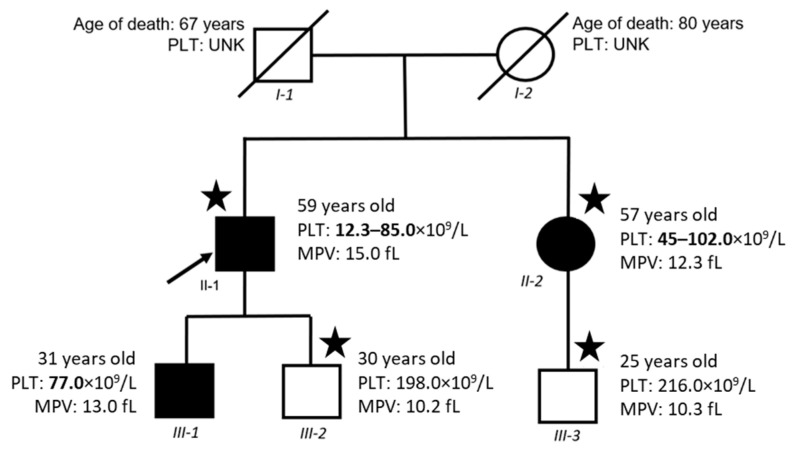
Proband’s (II1) family pedigree shows segregation of macrothrombocytopenia, platelet count (PLT), and mean platelet volume (MPV). Squares represent male, circles represent female individuals, black squares and circles mean macrothrombocytopenia. The samples with an asterisk were analyzed by whole-exome sequencing. UNK—unknown.

There were three patients suspected of inherited platelet function disorder (IPFD) and macrothrombocytopenia and two healthy individuals in the family investigated at the University Hospital Brno. Careful clinical evaluation of patients II-1 (proband), II-2, and III-1 (see Figure 1), including personal (healthy lifestyle) and familial bleeding history (unexplained bruising, epistaxis, menorrhagia, and bleeding during childbirth, and dental extractions) were obtained. Complete physical examination, such as hearing loss, heart, face, or bone dysmorphisms, ocular involvement, mental retardation, and skin discoloration was carried out. Furthermore, clinical assessment and a bleeding score were determined according to the bleeding questionnaire (Table S1) [16,17,18]. Total bleeding scores of the patients were evaluated as 6 (II-1), 5 (II-2), and 3 (III-1).

In 2015, patient II-1 was first examined when he was admitted for a partial ischemic stroke of the left arterial circulation with secondary hemorrhagic changes, aphasia, and hemiparesis. At admission, the patient surprisingly was suffering from serious thrombocytopenia (45 × 10^9^/L). The patient underwent intensive physiotherapy and logotherapy. He received low-molecular-weight heparin (LMWH) at a prophylactic dose. Subsequently, LMWH was switched to low-dose acetylsalicylic acid (ASA). In 6 months, this patient was admitted again with a second ischemic stroke of the posterior cerebral circulation with paresis of the right lower limb. Antiplatelet therapy was switched from ASA to clopidogrel. Currently, this patient has recovered partially from the aphasia and hemiparesis.

Regarding bleeding manifestations, patient II-1 suffered from repeated epistaxis with topical treatment in the past, bleeding from the gums only after brushing teeth, and prolonged post-traumatic bleeding. Unfortunately, light transmission aggregometry was first conducted after admission to the hospital on ASA treatment. In September 2017, the patient developed bradycardia (45/min) and paroxysm of atrial fibrilloflutter. Anticoagulant treatment was indicated. Further, a Boston Acclolade L311 DR pacemaker was implanted. For the next 3 months, the patient was treated with dual antiplatelet therapy and after that with ASA.

**Figure 2:**
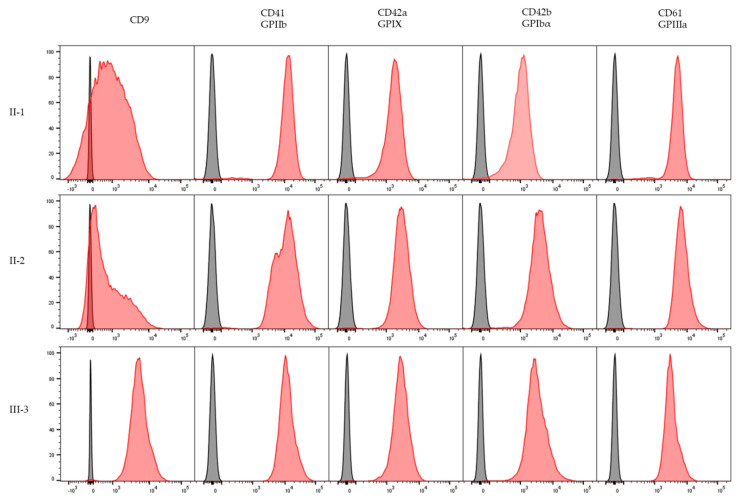
Platelet immunophenotype. CD41, CD61, CD42a, CD42b, CD9 antigens expression and negative controls on the platelets of two thrombocytopenic patients (II-1, II-2) and one healthy individual (III-3). Flow cytometric analysis shows decreased expression of the GPIbα-IX complex and CD9 and slightly increased expression of GPIIb and GPIIIa in both patients compared to the healthy individual. Negative controls are in grey, population of interest is in red. The peak height is normalized to mode.

Patient II-2 underwent splenectomy with no bleeding complications. Splenectomy was indicated for steroid-refractory thrombocytopenia (45 × 10^9^/L). Consequently, there was an improvement in the platelet count after the splenectomy. Patients II-1 and II-2 underwent preliminary laboratory investigation, including full blood count and prothrombin time. Platelet counts and volume were controlled by counting platelets visually in a light microscope according to standard procedures. Platelet aggregation was performed in light transmission aggregometry (LTA) and the analysis of major platelet surface glycoproteins by flow cytometry (Figure 2). Whole-exome sequencing (WES) analysis was performed in two patients with macrothrombocytopenia (II-1; II-2) and two healthy individuals (III-2, III-3). Sanger sequencing confirmed the WES results and the absence of a *GP1BA* gene variant in the healthy individuals III-2 and III-3. In addition, the *GP1BA* variant was found by Sanger sequencing in the III-1 individual. Healthy individual III-3 was used as a control sample for flow cytometric analysis and transmission electron microscopy (TEM). Immunoblot of the platelet fraction in patient II-1 verified decreased expression of GPIbα compared with healthy individual III-2. Pedigree analysis indicated an AD mode of inheritance (Figure 1).

### 2.2. Mutational Screening

WES identified a heterozygous variant within *GP1BA* exon 2 (NM_000173:c.98G > A:p.C33Y) in both affected family members with macrothrombocytopenia (II-1; II-2). The variant was not detected in the healthy individuals (III-2, III-3). The results were verified by Sanger sequencing. The heterozygous variant within *GP1BA* exon 2 (NM_000173:c.98G > A:p.C33Y) was also found in a third family member with macrothrombocytopenia (III-1) by Sanger sequencing.

First, the WES variants evaluation process was focused on the analysis of single nucleotide variants and short indels within the virtual panel of 363 platelet-associated genes (Table S4), where c.98G > A was characterized as the only “likely pathogenic” variant. In the next step, the evaluation of all WES data was performed, assuming the concordance of variants in both affected family members with regard to the mode of inheritance. Within the WES data, a total of 57 identical gene variants were identified in both patients, which were completely absent in healthy individuals (III-2, III-3) (Table S2). However, according to publications and databases, the detected gene variants, except for the *GP1BA* variant, have not yet been associated with inherited hematological diseases. Lastly, the presence of variants in 5’UTR of the *ANKRD26* gene was screened by Sanger sequencing in five family members (II-1, II-2, III-1, III-2, III-3) with negative results.

Recently, the single nucleotide substitution c.98G > A in the *GP1BA* gene was described in a Denmark family with diagnosed monoallelic BSS in three patients [10]. However, the variant c.98G > A is absent in ExAC, 1000 genomes, KAVIAR, gnomAD, HGMD databases, and Alamut^®^ Visual 2.14 Software (SOPHiA Genetics, Boston, MA, USA). Ma et al. [11] in April 2021 reported a single case of an 18-month-old Chinese girl diagnosed with monoallelic BSS carrying a heterozygous c.97T > A variant in *GP1BA* which led to substitution p.C33R.

**Figure 3:**
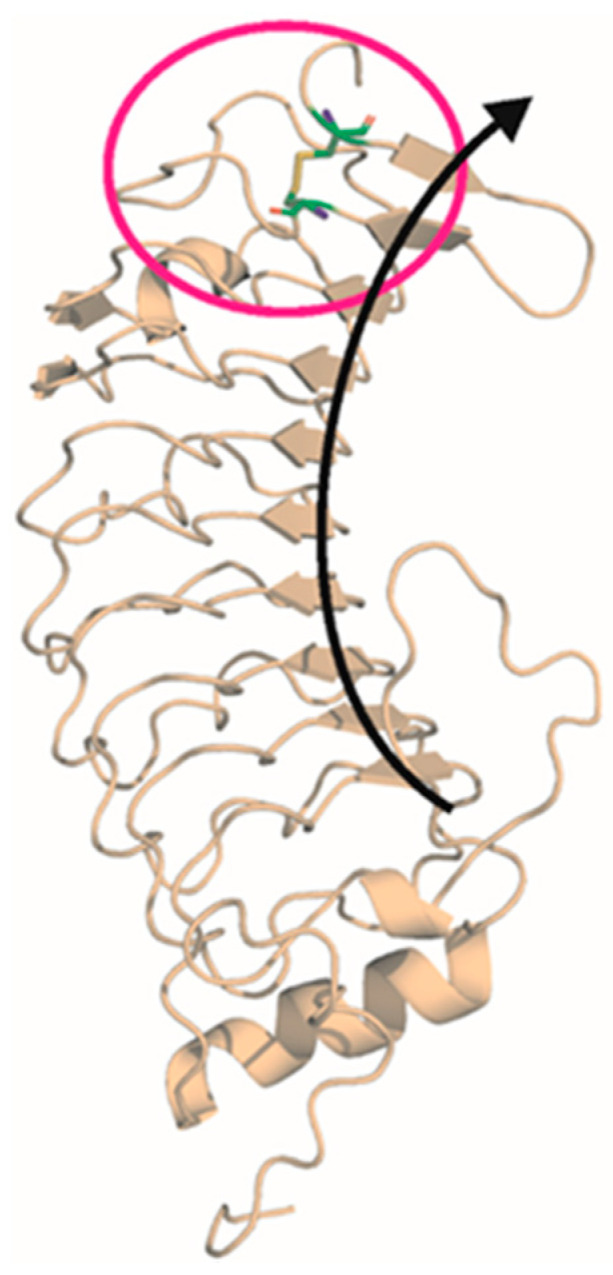
Human crystal structure of the N-terminal domain of GPIbα (pdb code 1p9a). Cys33 and Cys20 creating a disulfide bridge are highlighted in the magenta circle. Leucine-rich repeats folded into β-strands create an arc, which is highlighted by the black arrow. Cysteine–tyrosine substitution results in a disruption of a disulfide bond.

The *GP1BA* variant was located within the N-terminal leucine-rich repeat (LRR) domain of GPIbα. The N-terminal domain of GPIbα consists of eight leucine-rich-repeats (LRRs) made up of parallel β-strands, which folds into an arc (Figure 3). The presence of central LRRs was suggested to be important for the binding of VWF [19]. Based on the crystal structure of the N-terminal domain of the human physiologic GPIbα [20], in silico analysis showed a disulfide bridge between Cys33 and Cys20. Replacement of cysteine 33 with tyrosine will disrupt this bridge, which most probably induces disruption of the protein structure. It is to be noted that Cys33 and Cys20 are localized in the loops adjacent to the terminal β strands so they can contribute to the stability of the arc. Based on this knowledge, we presumed a pathogenic character of the p.C33Y variant.

### 2.3. Platelet Immunophenotype

Flow cytometric analyses of expression of CD41 (GPIIb), CD61 (GPIIIa), CD42b (GPIbα), CD42a (GPIX), and CD9 antigens in platelet-rich plasma (PRP) samples from the affected patients (II-1, II-2) and healthy individual (III-3) were performed. Platelet immunophenotype showed decreased expression of the GPIbα-IX complex as well as CD9 in the patients and slightly increased GPIIb and GPIIIa expression (Figure 2) which corresponded with mean fluorescence intensity (MFI) values (Table S3). On the contrary, expression of all measured CD antigens was normal in a healthy individual (III-3).

### 2.4. Platelet Aggregation Function

The proband was receiving long-term antiplatelet therapy with acetylsalicylic acid (ASA) due to previous strokes. New platelet aggregation tests of the proband (II-1) revealed hypoaggregation (60%) after ristocetin (1.5 mg/mL); however, there was also significant hypoaggregation after other platelet agonists: adenosine diphosphate (ADP, 5 µmol/L and 10 µmol/L), collagen (2 µg/mL and 5 µg/mL), and arachidonic acid (ARA, 1.5 mmol/L). The platelet aggregation function was normal in the healthy individual III-2 (Table 1). Original platelet aggregation tests (2017) of the proband (II-1) and his sister (II-2) revealed severe hypoaggregation (20%) after ristocetin (1.5 mg/mL); however, there was also significant hypoaggregation after other platelet agonists (Table S5).

### 2.5. TEM Confirmed Macrothrombocytopenia

**Figure 4:**
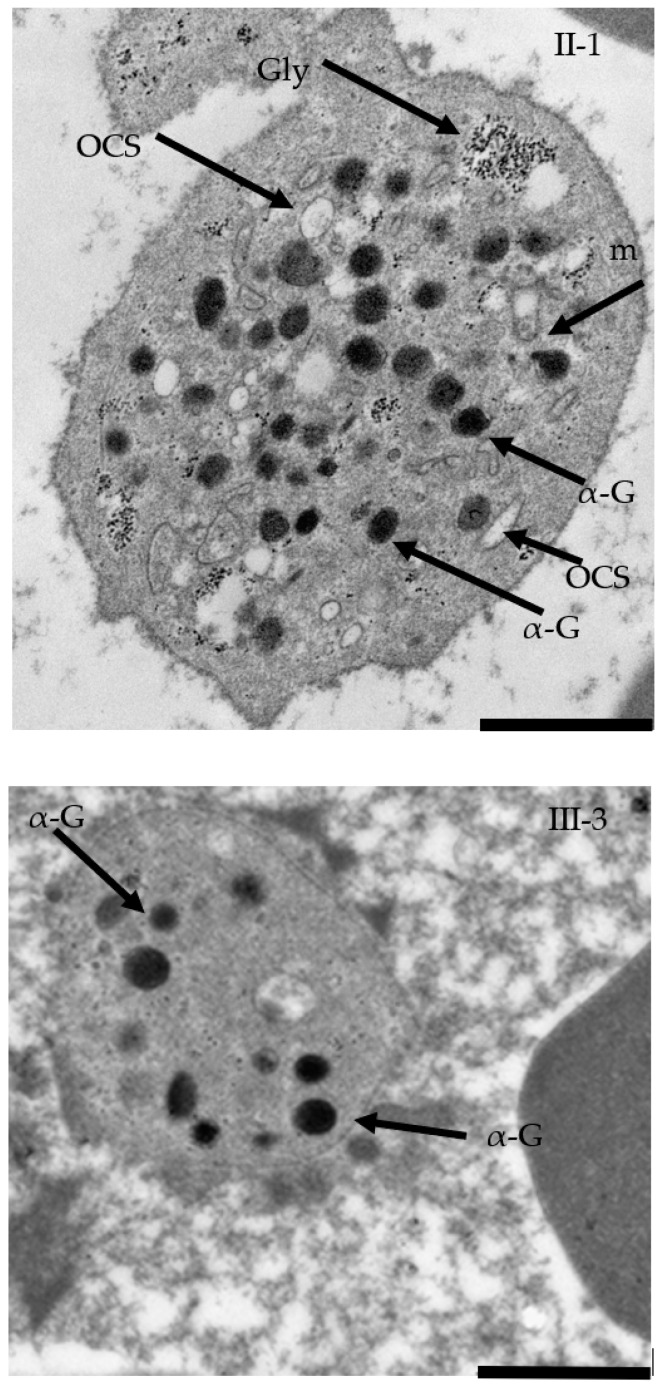
Transmission electron microscopy of a platelet from the affected individual (II-1) and the healthy individual (III-3) platelets. The platelet of the affected individual II-1 is enlarged, containing an increased number of α-granules (α-G) of different shapes. The surface-connected open canalicular system (OCS) and an intact mitochondrion (m) and glycogen (Gly) are also seen. Scale bar (a black bar bottom right) represents 1µm.

**Figure 5:**
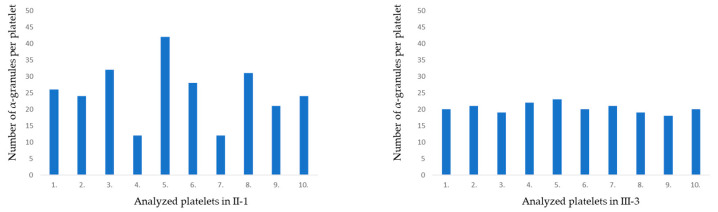
Histograms showing number of α-granules per platelet in patient II-1 and healthy individual III-3.

TEM confirmed the presence of macrothrombocytes (up to 4.0 µm in diameter) with an increased number of α-granules of different shapes in patient II-1 compared with the standard number of α-granules in the healthy individual III-3 (Figure 4). A histogram showing the number of α-granules per platelet was computed from 10 platelets (Figure 5). Mean and coefficient of variation (CV) were 25.2 ± 0.36 for patient II-1 and 20.3 ± 0.07 for healthy individual III-3; Mann–Whitney test (*p* < 0.045). Moreover, greater variability in the α-granules number was present in the II-1 platelets.

### 2.6. Immunoblot Analysis

**Figure 6:**
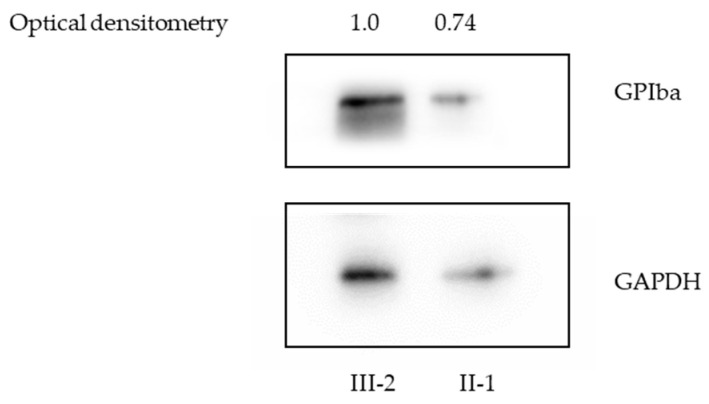
Immunoblot analysis of platelets proteins of patient II-1 and healthy individual III-2 showed decreased expression of GPIbα1ba in patient II-1 (0.74) compared with healthy individual III-2 (1.0). Antibody GPIba, Rabbit (1:3000), Sigma-Aldrich, antibody GAPDH, Rabbit (1:3000), Cell Signaling.

Immunoblot of platelets confirmed decreased expression of GPIbα in patient II-1 (0.74) compared with the healthy individual III-2 (1.0) (Figure 6).

## 3. Discussion

In this report, we present a family with macrothrombocytopenia and mild bleeding tendency. We report a germline heterozygous variant c.98G > A leading to substitution of p.C33Y in the *GP1BA* gene, segregating with macrothrombocytopenia in two generations. A causal heterozygous variant c.98G > A of mBSS was described by Leinøe et al. [10] in three patients with moderate thrombocytopenia (81–90 × 10^9^/L) and undefined macrothrombocytopenia. However, at the same amino acid position was detected the nucleotide variant c.97T > A (p.C33R) in a Chinese family with severe thrombocytopenia (31 × 10^9^/L), giant platelets, and normal platelet aggregation [11]. Furthermore, we have combined routine laboratory examination of patients with state-of-the-art genomic, flow cytometric, immunoblot, and microscopic approaches to understand the impact of the previously undescribed variant on the patients’ phenotype. More than 60 genetic variants in *GP1BA* have been associated with the classic AR BSS, but to date, only 14 variants have been associated with its AD form. Except for the p.A172V (Bolzano) and p.C20G (Copenhagen) variant [4,5,10], these variants were unique and family specific. Interestingly, all of these variants encoded amino-acid substitution within the LRR region of the GPIbα protein, exerting a dominant-negative effect on the protein structure. Previously described variants affect amino-acid residues deep within the protein structure, disrupting the folding of the LRR region and thus the formation of the GPIb-IX-V complex [1,3,4,5,6,7,8,9,10,13,14,15,20]. In the case of the here analyzed family, the detected variant substitutes cysteine residue for tyrosine in the conserved N-terminal flanking domain. This results in disruption of the Cys20-Cys33 disulfide bond, which stabilizes terminal LRRs within the arc structure. The importance of Cys33 for LRRs stability supports our hypothesis of the AD mode of inheritance of the p.C33Y variant. Using various in silico online prediction tools, we suggested the likely pathogenic effect of the discovered variant.

The decreased CD9 platelet expression results in the BSS patients are in concordance with the results from the Brazilian BSS patient’ cohort published by Beltrame et al. [21], where decreased expression of CD9 together with GPIbα and GPIX in BSS was first described. Decreased CD9 platelet expression in patients with BSS has also been described by Qiao et al. [22]. The observation that CD9 and GPIIb-GPIIIa are stored in the same intracellular structures and migrate to the same activation zones after platelet stimulation supports the previous suggestion of a close association between CD9 and GPIIb-GPIIIa in human platelets and of the possible involvement of CD9 in the adhesive functions of platelets [21]. It is hypothesized that CD9 modulates integrin αIIbβ3, the major platelet integrin involved in thrombus stability. CD9 also regulates platelet activation and aggregation and cell adhesion [23].

First platelets aggregation of the patient II-2 from 2017 confirmed BSS diagnosis with severe hypoaggregation after ristocetin and only mildly impaired aggregation after other agonists (ADP, collagen, ARA). New platelet aggregation tests of the proband revealed hypoaggregation (60%) after ristocetin (1.5 mg/mL); however, there was also significant hypoaggregation after other platelet agonists: ADP, collagen, and ARA. The results of these tests were affected by the ASA therapy in the proband. Another explanation for the observed hypoaggregation after all aggregation agonists in patients II-1 and II-2 could be the setting of reference limits, which were determined from a normal platelet count in PRP (150–350), while the platelet count in patients II-1 and II-2 were 105 and 102 for these tests. Clinical presentation of BSS patients is heterogeneous: some BSS patients have mainly macrothrombocytes with normal platelet function and may or may not have thrombocytopenia [24]. In addition, in patients who have thrombocytopenia, platelet aggregation may be altered [25]. In our case, we confirmed the monoallelic BSS phenotype in the patient (II-1) based on the platelet aggregation test.

We observed morphological differences of platelets between the affected and healthy family members. We confirmed the presence of macrothrombocytes with an increased number of α-granules (α-G) (25.2 ± 0.36) of different shapes in the II-1 patient (Figure 4) compared to the healthy individual III-3 (20.3 ± 0.07). Moreover, our data also demonstrated greater variability in α-granule numbers between samples II-1 and III-3, which further supports our hypothesis of likely pathogenic variants. α-granules are essential to normal platelet activity. These unusual secretory granules derive their cargo from both regulated secretory and endocytic pathways in megakaryocytes. Furthermore, defects in α-granule formation have been described in some inherited disorders of α-granules formation, but not in the BSS diagnosis. Functional roles of platelet α-granules are derived from their contents and are associated with platelet adhesion. Components of the VWF complex, GPIbα-IX-V were found in α-granules [26]. The increased number of α-granules in this presented case might be explained by the supposition that the GPIbα protein is expressed but does not reach the platelet surface. However, the precise mechanism remains unclear.

Immunoblot of platelets revealed decreased expression of the GPIbα protein in proband II-1 compared with the healthy individual (III-2). This abnormality of the expression of glycoprotein Ibα of the p.C33Y variant confirms the likely pathogenic effect of the discovered variant.

According to Leinøe et al. [10], the GPIbα-IX-V receptor membrane complex plays a pivotal role in thrombosis. Thus, a reduction in the expression of GPIbα-IX-V in mBSS patients may protect against arterial thrombosis and thereby promote a survival advantage. Here, we report the mBSS patient (II-1) with two previous ischemic strokes despite heterozygous *GP1BA* variant and reduction of GPIbα-IX-V complex expression. (Mother (I-2) of this patient had five ischemic strokes before she died, but the *GP1BA* status is due to the lack of DNA sample unknown). In the here presented patient II-1, the reduced expression of GPIbα-IX-V seems not to be protective enough against arterial thrombosis, but other cases are needed for a general conclusion.

In conclusion, the described *GP1BA* gene variant causes changes at the protein level, which may affect its protein conformation, expression, and thereby disturb the binding to its interacting partners and cause macrothrombocytopenia with decreased expression of GPIbα, GPIIa, GPIIIb, CD9 in this case of monoallelic BSS (AD macrothrombocytopenia). Our report also points to the complexity and difficulty of functional analyses and their interpretation in similar cases.

## 4. Materials and Methods

### 4.1. Study Approval

Patients were recruited from University Hospital Brno after written informed consent was obtained, in accordance with the Declaration of Helsinki and protocols approved by the institutional ethics committees.

### 4.2. Mutational Screening

We performed whole-exome sequencing of four family members (II-1, II-2, III-2, III-3), as shown in Figure 1. Samples of peripheral blood were collected and processed for genomic DNA isolation using the MagCore^®^ Genomic DNA Whole Blood Kit (RBC Bioscience, UK). Whole-exome libraries were processed using the KAPA Hyper Prep Kit, SeqCap EZ MedExome Enrichment Kit, and HyperCap Bead Kit (Roche, Pleasanton, CA, USA) according to the SeqCap EZ HyperCap Workflow v2.1 following the recommended protocols. Paired-end 2x75 bp sequencing was performed on the Illumina NextSeq 500 Sequencer (Illumina Inc., San Diego, CA, USA). The sequencing data met the set QC standards for our department: 90% of reads were mapped to the regions of interest, which had a coverage ≥30×. The FastQC tool was applied for quality checks of the sequenced samples. The raw sequencing reads were aligned to the GRCh38 reference genome using BWA-mem, version 0.7.15, including polymerase chain reaction duplicate marks. Germline single nucleotide variants (SNV) and indels were detected via local re-assembly of haplotypes in the GATK HaplotypeCaller, version 3.7. Annotation of the obtained variants/indels was performed with Annovar. The processed SNV/indels were compared first in a panel of 363 platelet-associated genes (Table S4) and then matched to the variants within a whole exome [27]. To identify clinically relevant SNV/indels, only variants with total coverage of at least 15×, minor allele frequency (MAF) values ≤ 0.01, and predicted possible-probable deleteriousness were included.

Finally, the classification of the characterized variant p.C33Y in the *GP1BA* gene was determined and called using the consensus guidelines as set out by the American College of Medical Genetics and Genomics and the Association for Molecular Pathology (ACMG/AMP guidelines). The segregation of the p.C33Y variant in four family members was determined by Sanger sequencing using a BigDye Terminator v3.1 Cycle Sequencing Kit according to the manufacturer’s protocol. The primers were designed for the detection of exon 2 in the *GP1BA* gene (F-primer: AGGGGGATCCACTCAAGGC, R-primer: GTCCCATCGACCTGGAGC). Capillary sequencing was performed using BigDye-terminator chemistry on the 3500 Genetic Analyzer (Applied Biosystems, Warrington, UK) [28,29,30,31,32,33].

### 4.3. In Silico Analysis

The deleterious effect of the p.C33Y was evaluated using in silico online prediction tools according to Table 2. In addition, the crystal structure [20] of the N-terminal domain of human platelet receptor glycoprotein Ib-alpha (GP Ibα, pdb code 1p9a) was analyzed using the VMD program [34] to better understand the mutation’s effect at the protein structure level.

### 4.4. Platelet Immunophenotype

Peripheral blood samples of the affected patients (II-1, II-2) and a healthy individual (III-3) were collected into EDTA-coated tubes, and PRP was prepared by centrifugation. PRP samples were stained with a single or tricolor combination of fluorochromes. Monoclonal antibodies included CD9: a member of the tetraspanin superfamily (M-L13, Becton, Dickinson and Company, Franklin Lakes, NJ, USA), CD41:GP IIb (VIPL3, Invitrogen), CD42a:GP IX (SZ1, Beckman Coulter), CD42b:GP Ibα (MB45, Invitrogen, ThermoFisher Scientific, Waltham, MA, USA), and CD61:GP IIIa (PM6/13, Fitzgerald Industries International, Acton, MA, USA). An unlabeled negative control sample (native PRP sample) was also prepared. After incubation with antibodies, the samples were analyzed on the flow cytometer BD FACS CANTO II (Becton Dickinson, Franklin Lakes, NJ, USA).

### 4.5. Platelet Aggregation Tests

In vitro platelet aggregation was studied in citrated platelet-rich plasma. The platelet-rich plasma was obtained by centrifugation of blood using the standard technique (at 200 *g* for 10 min). The platelet agonists were: collagen 2 µg/mL and 5 µg/mL (Hyphen BioMed, Neuville-sur-Oise, France), adenosine diphosphate (ADP, Hyphen BioMed, Neuville-sur-Oise, France) 5 µmol/L and 10 µmol/L, ristocetin (Hyphen BioMed, Neuville-sur-Oise, France) 1.5 mg/mL and low 0.75 mg/mL, arachidonic acid (ARA, Hyphen BioMed, Neuville-sur-Oise, France) 1.5 mmol/L at a final concentration. We performed aggregation tests on two family members (II-1 and III-2). The reference limits and maximum aggregation results in patients II-1 and III-2 were obtained (Table 1, Table S5). Reference aggregation limits were obtained from 50 blood donors. The reference limits were determined based on the results of the 2.5–97.5 percentile.

### 4.6. Electron Microscopy

TEM was used to analyze ultrastructural changes in platelets in our family: Venous blood drawn from the patients was mixed with acid citrate dextrose anticoagulant in a 6 to 1 ratio and PRP was prepared by centrifugation at 150 g for 20 min at room temperature. After fixation in 3% glutaraldehyde in phosphate buffer, the platelets were washed with phosphate buffer. Post fixation with 1% osmium tetroxide was performed and the specimens were dehydrated in a graded series of alcohol and embedded in LR white acrylic resins. Ultrathin serial sections were cut on a Leica Ultracut R ultramicrotome (Leica, Wetzlar, Germany), stained with uranyl acetate and lead citrate, and examined by a FEI Tecnai G2 F20 microscope (ThermoFisher Scientific, Waltham, MA, USA).

### 4.7. *Immunoblotting*

Peripheral blood samples of the affected patient (II-1) and the healthy individual (III-2) were collected into sodium citrate coated tubes, platelet isolation was prepared by the density gradient centrifugation and washing method [35]. The platelets sample of the affected patient (II-1) contained: 2.3% leukocytes (CD45), 84.0% thrombocytes (CD41, CD61, CD9), and the healthy individual (III-2) contained: 0.86% leukocytes and 94.3% of thrombocytes (CD41, CD61, CD9) according to flow cytometric analysis. Immunoblotting of platelets proteins of patient II-1 and the healthy individual III-2 were diluted with Tris-SDS buffer (5 mM Tris, 1% SDS, 10% glycerol) and proceeded the same as follows: 10 µg of protein lysates were loaded per well. The samples were resolved on 10% SDS-PAGE and blotted to the PDVF membrane. The membranes were labeled with anti-GPIba (1:3000), Rb, Sigma, anti-GAPDH (1:3000), Rb, Cell Signaling antibodies combined with the Clarity Western ECL Substrate (Bio-Rad, Hercules, CA, USA). The chemiluminescence image of proteins was acquired on the UVITEC CAMBRIDGE system. Optical densitometry of protein bands was calculated using FiJi (ImageJ v.1.53c, open-source software).

## Figures and Tables

**Table 1:** The reference limits and maximum aggregation results in patient II-1 and healthy individual III-2.

Name of Agonists	Reference Limits	Patient II-1	Healthy Individual III-2
Platelet count in PRP (×10^9^/L)	150–300	105	228
Platelet count in PPP (×10^9^/L)	0–20	0	1
Aggregation—collagen			
Collagen 2—Amax (%)	74.5–87.3	57.9	79.9
Collagen 5—Amax (%)	74.7–88.9	72	75.8
Aggregation—ADP			
ADP 5—Amax (%)	57.0–86.2	57.5	69.2
ADP 5—disaggregation (%)	0–10.0		
ADP 10—Amax (%)	66.6–90.7	64.1	69.1
ADP 10—disaggregation (%)	0–10.0		
Aggregation—ristocetin			
Ristocetin—Amax (%)	77.8–97.1	60.6	84.9
Ristocetin correction—Amax(%)	N.A.		36.4
Low ristocetin—Amax (%)	0.0–10.0	1.0	2.6
Aggregation—arachidonic acid			
Arachidonic acid—Amax (%)	73.2–89.6	4.7	75.0
Spontaneous aggregation	less than 5	N.D.	N.D.

The reference limits were determined based on the results of the 2.5–97.5 percentile. Reference aggregation limits were obtained from 50 peripheral blood donors. Not applicable N.A., not done N.D.

**Table 2:** In silico online prediction tools.

Tool	Link	Score and Limits	Interpretation
Align GVGD	http://agvgd.hci.utah.edu/agvgd_input.php	C65	“most likely pathogenic”
MetalR	google.com/site/jpopgen/dbNSFP	0.9997 (>1.0)	“to be deleterious”
MutationTaster	http://www.mutationtaster.org	1.00 (≥0.46)	“disease-causing”
PROVEAN	http://provean.jcvi.org/index.php	−10.07 (≤−2.5)	“deleterious effect”
SIFT	http://provean.jcvi.org/index.php	0.001 (≤0.78)	“to be damaging”

## Data Availability

All data presented in this study are contained within this article or its supplementary materials. Further details are available upon request from the corresponding author.

## Supplementary Materials

The following are available online at https://www.mdpi.com/article/10.3390/ijms23020885/s1.
