# Supplementary material for: A GP1BA Variant in a Czech Family with Monoallelic Bernard-Soulier Syndrome
Source: Int J Mol Sci. 2022 Jan 14;23(2):885. doi: 10.3390/ijms23020885 (PMC8777725; doi:10.3390/ijms23020885)
Supplement: Supplementary file 1 [file ijms-23-00885-s001.zip › ijms-1511540-supplementary.pdf]

Supplementary Material

Table S1. Bleeding questionnaire and bleeding scores for the patients II-1, II-2, and III-1 with BSS according to [17].

Symptom	-1	0	1	2	3	4	Score II-1	Score II-2	Score III-1
Epistaxis	-	No or trivial (<5)	>5 OR more than 10′	Consultation only	Packing or cauterization or antifibrinolytics	Blood transfusion or replacement therapy	3	1	1
Cutaneous	-	No or trivial (<1cm)	>1cm AND no trauma	Consultation only			N/A	N/A	N/A
Bleeding from minor wounds	-	No or trivial (<5)	> 5 OR more than 5'	Consultation only or sterile strips	Surgical hemostasis or antifibrinolytics	Blood transfusion or replacement therapy	1	1	1
Oral Cavity	-	No	Reported at least one	Consultation only	Surgical hemostasis or antifibrinolytics	Blood transfusion or Replacement therapy	1	1	N/A
GI Bleeding	-	No	Identified cause	Consultation or Spontaneous	Surgical hemostasis, antifibrinolytics, blood transfusion	Replacement therapy	N/A	N/A	N/A
Tooth extraction	No bleeding in at least 2 extractions	None done, No bleeding in 1	Reported, no consultation	Consultation only	Resuturing, repacking or antifibrinolytics	Blood transfusion or Replacement therapy	1	1	1
Surgery	No bleeding in 2 surgeries	None done, No bleeding in 1	Reported, no consultation	Consultation only	Surgical hemostasis or antifibrinolytics	Blood transfusion or Replacement therapy	N/A	N/A	N/A
Menorrhagia	-	No	Reported, or consultation only	Antifibrinol ytics or Pill use	D&C, Iron therapy	Blood transfusion or Replacement therapy or Hysterectomy	N/A	1	N/A
Post-partum hemorrhage	No bleeding in at least 2 deliveries	No deliveries, No bleeding in 1 delivery	Reported, or consultation only	D&C, Iron therapy, Antifibrinol ytics	Blood transfusion or replacement therapy	Hysterectomy	N/A	N/A	N/A
Muscle hematomas	-	Never	Post trauma no therapy	Spontaneous no therapy	Spontaneous or traumatic, requiring replacement therapy	Spontaneous or traumatic, requiring surgical intervention or blood transfusion	N/A	N/A	N/A
Hemarthrosis	-	Never	Post trauma no therapy	Spontaneous no therapy	Spontaneous or traumatic, requiring replacement therapy	Spontaneous or traumatic, requiring surgical intervention or blood transfusion	N/A	N/A	N/A
CNS Bleeding	-	Never	-	-	Subdural, any intervention	Intracerebral, any intervention	N/A	N/A	N/A
-	-	-	-	-	-	**Bleeding score:**	**6**	**5**	**3**

Not Available (N/A)

Table S2. Heterozygous gene variants from WES analysis filtered for the macrothrombocytopenia phenotype. Reference genome: GRCh38; variants and indels were filtered for coding regions and splice sites; minimal coverage 15×. These gene variants were identified in both patients (II-1, II-2), which were completely absent in healthy individuals (III-2, III-3).

Genomic coordinate	Gene	RefSeq	cDNA	Protein	II-1 VAF [%]	II-2 VAF [%]
62728902	*KANK4*	NM_001320269	c.517T>C	p.Y173H	52.94	40.00
145608556	*POLR3C*	NM_001303456	c.290G>A	p.R97Q	39.02	35.29
148004763	*NBPF26*	NM_001351372	c.3796G>A	p.E1266K	41.07	46.15
153012650	*SPRR2D*	NM_006945	c.173C>T	p.T58I	41.03	55.56
153430347	*S100A7*	NM_002963	c.241G>T	p.D81Y	58.62	58.33
156106994	*LMNA*	NM_001282625	c.1579C>T	p.R527C	93.40	55.38
161332307	*SDHC*	NM_001035511	c.430G>C	p.E144Q	37.93	48.84
201179738	*IGFN1*	NM_001164586	c.5717C>A	p.A1906E	46.67	30.77
43768308	*THADA*	NM_001345925	c.3254C>T	p.T1085M	50.00	42.50
54120827	*PSME4*	NM_014614	c.4022G>A	p.R1341H	62.86	37.50
54155315	*PSME4*	NM_014614	c.1442T>C	p.M481T	43.82	50.00
152404198	*NEB*	NM_001164507	c.20212G>A	p.D6738N	63.04	55.88
152404248	*NEB*	NM_001164507	c.20162T>C	p.L6721P	56.76	57.14
152530992	*NEB*	NM_001164507	c.3986A>C	p.D1329A	50.00	43.55
160634452	*CD302*	NM_001198759	c.5416T>C	p.S1806P	31.25	56.25
186653566	*FSIP2*	NM_173651	c.1703A>T	p.Y568F	51.14	61.54
186665996	*FSIP2*	NM_173651	c.11963C>T	p.S3988F	43.24	52.73
13600704	*BOD1L1*	NM_148894	c.7820A>T	p.N2607I	50.91	36.00
47574926	*ATP10D*	NM_020453	c.3278A>G	p.K1093R	47.06	30.43
62812764	*ADGRL3*	NM_001322402	c.2552C>T	p.T851M	54.43	43.75
88084724	*KLHL8*	NM_001292003	c.1807_1810del	p.T603fs	46.30	51.61
108868556	*CYP2U1*	NM_183075	c.1151G>T	p.R384I	51.46	44.44
155533337	*FGG*	NM_000509	c.140C>T	p.T47I	39.02	60.71
54786796	*PLPP1*	NM_176895	c.205A>G	p.I69V	77.78	46.88
74412411	*ANKRD31*	NM_001164443	c.3964T>C	p.S1322P	54.10	43.33
79616698	*SPZ1*	NM_032567	c.664G>A	p.E222K	45.83	50.00
151180378	*G3BP1*	NM_198395	c.1142G>A	p.G381D	37.84	46.81
42858231	*C6orf226*	NM_001008739	c.296T>G	p.V99G	39.13	30.77
42931129	*GNMT*	NM_018960	c.658A>T	p.T220S	47.73	39.29
76023429	*FILIP1*	NM_001289987	c.2127_2128insGG	p.R710fs	55.21	52.27
17379805	*AHR*	NM_001621	c.2356A>G	p.M786V	47.37	52.17
141813647	*PTK2*	NM_005607	c.1036C>T	p.P346S	47.22	41.46
144810414	*FAM83H*	NM_198488	c.1217C>A	p.A406D	43.75	39.13
134351730	*PRRC2B*	NM_013318	c.4214T>G	p.L1405R	40.00	69.23
139265345	*CARD9*	NM_052814	c.575A>G	p.Q192R	59.09	50.00
96162517	*TBC1D12*	NM_015188	c.147_164del	p.49_55del	46.15	30.77
3141665	*OSBPL5*	NM_020896	c.592G>A	p.V198I	60.47	42.86
7712604	*OVCH2*	NM_198185	c.1535C>G	p.T512S	51.85	53.85
10862595	*YBX3*	NM_003651	c.692A>C	p.Q231P	60.71	40.00
15777273	*EPS8*	NM_004447	c.2113G>A	p.A705T	45.45	57.14

26217934	*RASSF8*	NM_001164747	c.607G>C	p.E203Q	51.43	48.89
96379884	*HAL*	NM_001258334	c.1106G>A	p.R369Q	53.85	50.00
123057763	*KNTC1*	NM_014708	c.2214G>C	p.E738D	52.54	51.72
**4835997**	***GP1BA***	**NM_000173**	**c.98G>A**	**p.C33Y**	**55.00**	**60.00**
74003318	*EVPL*	NM_001320747	c.6034C>T	p.R2012W	64.71	40.74
78306001	*RNF213*	NM_001256071	c.3713G>A	p.R1238Q	47.27	68.29
7049171	*LAMA1*	NM_005559	c.674G>A	p.R225H	47.06	39.66
8825397	*MTCL1*	NM_015210	c.3889T>C	p.S1297P	41.38	50.00
8825398	*MTCL1*	NM_015210	c.3890C>T	p.S1297F	39.29	51.35
54423856	*WDR7*	NM_015285	c.2032G>T	p.A678S	34.21	50.00
17212814	*MYO9B*	NM_001130065	c.287A>G	p.Q96R	42.22	53.85
18980866	*GDF1*	NM_001492	c.251G>A	p.G84E	50.00	50.00
19033544	*DDX49*	NM_019070	c.767A>G	p.N256S	41.38	60.00
43990796	*PHLDB3*	NM_198850	c.1393G>A	p.A465T	39.39	54.84
53116748	*ZNF83*	NM_001348019	c.1070A>G	p.N357S	50.91	53.33
53912045	*ZNF765*	NM_001040185	c.1237T>G	p.C413G	34.25	39.47
42315606	*MYBL2*	NM_002466	c.394G>C	p.E132Q	48.75	60.00
153220494	*HCFC1*	NM_005334	c.3270_3356del	p.1090_1119del	48.65	56.25

VAF – variant allele frequency

Table S3. Mean fluorescence intensity of antigens expression on platelets in patients II-1, II-2 and one healthy individual III-3.

**MFI**	**II-1**		**II-2**		**III-3**	
FSC	4566		7118		4480	
CD41	14431	99.3%	13253	99.3%	12656	99.6%
CD61	4886	99.3%	7468	99.3%	3406	99.6%
CD42a (GPIX)	1814	98.9%	3005	98.9%	3803	99.6%
CD42b (GPIbα)	1113	98.5%	4287	98.9%	4622	99.4%
CD9	1236	50.4%	1082	54.1%	5560	99.6%

Mean fluorescence intensity (MFI), forward scatter (FSC); reference limits for percentage values (in-house reference level): CD41- 80-99%, CD61- 75-99%, CD42a- 66-99%, CD42b- 70-98%, cut-of limit for MFI >100.

Table S4. Virtual panel of inherited thrombocytopenia related genes based on the literature review [27].

ABCA12	*BLOC1S2*	*FHOD1*	*ITGA2*	*MYO3A*	*PPP1CB*	*RASGRP2*	*STXBP4*
ABCB4	*BLOC1S3*	*FLI1*	*ITGA2B*	*MYO5A*	*PPP1CC*	*RBM8A*	*STXBP5L*
ABCC4	*BLOC1S4*	*FLII*	*ITGA5*	*MYO5B*	*PPP1R12A*	*RGS10*	*STXBP6*
ABCG5	*BLOC1S5*	*FLNA*	*ITGB1*	*NAPA*	*PPP1R12C*	*RGS18*	*SUZ12*
ABCG8	*BLOC1S6*	*FMNL1*	*ITGB3*	*NAPG*	*PPP1R14A*	*RGS19*	*SYK*
ACSL4	*BMP4*	*FMNL3*	*ITPR1*	*NBEA*	*PPP1R2*	*RGS20*	*SYTL3*
ACTN1	*BTBD9*	*FRMPD1*	*JAK2*	*NBEAL2*	*PRKACA*	*RGS9*	*SYTL4*
ACVRL1	*BTK*	*FYB*	*JMJD1C*	*NFE2*	*PRKACB*	*RHOA*	*TAL1*
ADAMTS13	*C14orf133*	*FYN*	*KIAA1109*	*NIPSNAP3A*	*PRKACG*	*RHOC*	*TAOK1*
ADCY3	*C19orf55*	*GATA1*	*KIAA2018*	*NOTCH1*	*PRKAR1A*	*RHOF*	*TBXA2R*
ADCY6	*C20orf42*	*GDI2*	*LAIR1*	*NOX1*	*PRKAR2A*	*ROCK1*	*TEC*
ADCY7	*C6orf25*	*GFI1*	*LAT*	*NRG3*	*PRKCA*	*ROCK2*	*TGFBR3*
ADORA2B	*CD226*	*GFI1B*	*LCP2*	*NSF*	*PRKCB*	*RUNX1*	*THPO*
ADRA2A	*CD36*	*GNA12*	*LPAR1*	*NXF1*	*PRKCD*	*SCAMP2*	*TLN1*
ADRA2B	*CLEC1B*	*GNA13*	*LTBP1*	*ORAI1*	*PRKCQ*	*SCAMP5*	*TLR2*
ADRBK1	*CLEC4F*	*GNAI1*	*LY6G6F*	*P2RX1*	*PRKD1*	*SCFD1*	*TMCC2*
AK3	*CNO*	*GNAI2*	*LYN*	*P2RY1*	*PRKG1*	*SELP*	*TPM1*
AKT1	*CSK*	*GNAQ*	*LYST*	*P2RY12*	*PRKG2*	*SERPINE2*	*TPM4*
AKT2	*CTTN*	*GNAZ*	*MAP2K2*	*P2RY13*	*PTEN*	*SH2B3*	*TRAF4*
ALOX12	*CYCS*	*GNB2*	*MAP2K4*	*PADI2*	*PTGIR*	*SIRPA*	*TREML1*
ANKRD12	*DAAM1*	*GNB3*	*MAP3K9*	*PDE2A*	*PTGS1*	*SLC35D3*	*TRPM7*
ANKRD18A	*DIAPH1*	*GNE*	*MAPK1*	*PDE3A*	*PTK2*	*SLC9A3R1*	*TTC37*
ANKRD18B	*DIAPH2*	*GNG11*	*MAPK13*	*PDE4D*	*PTPN1*	*SLC9A3R2*	*TTF2*
ANKRD26	*DIAPH3*	*GNG12*	*MAPK14*	*PDE5A*	*PTPN11*	*SLFN14*	*TUBA3C*
ANKRD33	*DNAH11*	*GNG13*	*MAPK8*	*PDPK1*	*PTPN12*	*SMAD1*	*TUBB1*
AP3B1	*DNM1L*	*GNG5*	*MDS1*	*PDZD3*	*PTPN18*	*SMAD6*	*UNC13A*
AP3D1	*DNM2*	*GP1BA*	*MECOM*	*PDZK1*	*PTPN2*	*SNAP23*	*UNC13B*
AP3M1	*DNM3*	*GP1BB*	*MKL1*	*PEAR1*	*PTPN6*	*SNAP25*	*VAMP2*
AP3S1	*DTNBP1*	*GP5*	*MLK1*	*PECAM1*	*PTPN7*	*SNAP29*	*VAMP3*
APC	*EFNB1*	*GP6*	*MLPH*	*PGM3*	*PTPN9*	*SNAPIN*	*VAMP7*
ARHGAP1	*EPHA4*	*GP9*	*MMP17*	*PHOX2A*	*PTPRA*	*SNX1*	*VAMP8*
ARHGAP17	*EPHB1*	*GRAP2*	*MNX1*	*PIK3CA*	*PTPRC*	*SRA1*	*VAV1*
ARHGAP32	*ERG*	*GRB2*	*MPL*	*PIK3CB*	*PTPRJ*	*SRC*	*VAV2*
ARHGAP6	*ETS1*	*GRK5*	*MRPS34*	*PIK3CD*	*RAB27A*	*SRF*	*VAV3*
ARHGDIA	*ETV6*	*GRK6*	*MUC16*	*PIK3CG*	*RAB27B*	*STIM1*	*VPS11*
ARHGDIB	*EXOC1*	*GUCY1A3*	*MUC2*	*PIK3R1*	*RAB38*	*STOM*	*VPS16*

ARHGEF12	*F2R*	*GUCY1B3*	*MUTED*	*PIK3R3*	*RAB4A*	*STX11*	*VPS18*
ARHGEF3	*F2RL3*	*HBB*	*MYB*	*PIK3R5*	*RABGGTA*	*STX12*	*VPS33A*
ARRB1	*FARP2*	*HOOK3*	*MYH10*	*PLA2G4A*	*RAC1*	*STX2*	*VPS33B*
ASPN	*FCER1G*	*HOXA11*	*MYH13*	*PLA2G4C*	*RAF1*	*STX4*	*VPS39*
BAK1	*FCGR2A*	*HPS1*	*MYH9*	*PLCB2*	*RAI1*	*STX6*	*VPS41*
BCL2L1	*FERMT1*	*HPS4*	*MYL9*	*PLCB3*	*RAP1B*	*STX7*	*VPS4B*
BCOR	*FERMT3*	*HTR2A*	*MYLK*	*PLCG2*	*RAP1GAP*	*STXBP1*	*VPS52*
BET1L	*FGD3*	*CHD3*	*MYLK2*	*PLDN*	*RAP1GAP2*	*STXBP2*	*VPS8*
BLOC1S1	*FGR*	*INPP5D*	*MYO18B*	*PPP1CA*	*RAP1GDS1*	*STXBP3*	*VWF*
					ZFPMI	*WDR66*	*WAS*

Table S5. The reference limits and maximum aggregation results in the patients II-1 and II-2 (2017).

Name of agonists	Reference limits	Patient II-1	Patient II-2
Platelet count in PRP (* 10^9/L)	150-300	58	102
Platelet count in PPP (* 10^9/L)	0-20	0	1
**Aggregation - collagen**			
Collagen 2 - Amax (%)	74.5 - 87.3	23.7	71.5
Collagen 5 - Amax (%)	74.7 - 88.9	45.3	75.8
**Aggregation - ADP**			
ADP 5 - Amax (%)	57.0 - 86.2	28	48.9
ADP 5 - disaggregation (%)	0 - 10.0		
ADP 10 - Amax (%)	66.6 – 90.7	31.3	69.1
ADP 10 - disaggregation (%)	0 - 10.0		
**Aggregation - ristocetin**			
Ristocetin - Amax (%)	77.8 – 97.1	20.2	20.3
Ristocetin correction - Amax(%)	N.A.	39.5	36.4
Low ristocetin - Amax (%)	0.2 - 4.7	1.4	2.5
**Aggregation - arachidonic acid**			
Arachidonic acid - Amax (%)	73.2 - 89.6	6.7	71.5
Spontaneous aggregation	less than 5	N.D.	N.D.

The reference limits were determined based on the results of the 2.5 - 97.5 percentile. Reference aggregation limits were obtained from 50 peripheral blood donors. Not applicable N.A., not done N.D.
